# Thirty Years of Cancer Nanomedicine: Success, Frustration, and Hope

**DOI:** 10.3390/cancers11121855

**Published:** 2019-11-25

**Authors:** Lucia Salvioni, Maria Antonietta Rizzuto, Jessica Armida Bertolini, Laura Pandolfi, Miriam Colombo, Davide Prosperi

**Affiliations:** 1 Department of Biotecnology and Bioscience, University of Milano-Bicocca, piazza della Scienza 2, 20126 Milano, Italy; lucia.salvioni@unimib.it (L.S.); maria.rizzuto@unimib.it (M.A.R.); jessica.bertolini@unimib.it (J.A.B.); miriam.colombo@unimib.it (M.C.); 2 Unit of Respiratory Diseases, IRCCS Policlinico San Matteo Foundation, 27100 Pavia, Italy; l.pandolfi@smatteo.pv.it; 3 Nanomedicine Laboratory, ICS Maugeri, via S. Maugeri 10, 27100 Pavia, Italy

**Keywords:** cancer nanomedicine, EPR effect, tumor microenvironment, nanoparticles, nano–bio interactions, clinical translation

## Abstract

Starting with the enhanced permeability and retention (EPR) effect discovery, nanomedicine has gained a crucial role in cancer treatment. The advances in the field have led to the approval of nanodrugs with improved safety profile and still inspire the ongoing investigations. However, several restrictions, such as high manufacturing costs, technical challenges, and effectiveness below expectations, raised skeptical opinions within the scientific community about the clinical relevance of nanomedicine. In this review, we aim to give an overall vision of the current hurdles encountered by nanotherapeutics along with their design, development, and translation, and we offer a prospective view on possible strategies to overcome such limitations.

## 1. Introduction

Nanomedicine is an emerging key technology of the 21st century. Although the fundamental concept of a new era of nanotechnology dates back to 1959 with the renowned visionary speech of Richard Feynman at Caltech [1], the optimistic expectation that nanoparticles and other nanoscale tools could be successfully exploited to improve the diagnosis and pharmacological treatment of several human diseases was only first established in the 1990s [2]. During the last three decades, we have witnessed impressive advances in the field, and our scientific understanding of the mechanisms regulating matter organization and interaction with biological systems at the nanoscale has progressed significantly. Nanomedicine, taking advantage of the use of engineered particles having size typically ranging from 1 to 100 nm, aims to exploit nanotechnology for several biomedical applications, mainly disease treatment, diagnosis, and molecular imaging, as well as regenerative medicine and tissue engineering. From the beginning, nanomedicine has been frequently associated with the use of nanoparticles in oncology [3].

In 1986, Maeda and coworkers observed a substantial accumulation of macromolecules in the tumor tissue attributable to a hyperpermeable neovasculature and compromised lymphatic drainage [4]. In principle, the fenestrated endothelial wall in proximity to tumor tissues represents a sort of privileged gate giving selective access to particles in the sub-micrometer scale. Since then, the so-called enhanced permeability and retention (EPR) effect has been validated for particles up to 400–600 nm [5], becoming the pillar of the research in cancer nanomedicine [6]. The general purpose was to improve the performance of chemotherapeutics, both in terms of efficacy and safety. These efforts resulted in the approval of several innovative nanodrugs and still inspire ongoing investigations [7]. However, after 30 years of exciting discoveries, together with the progress in clinical exploitation, several challenges and limitations are now emerging. Notably, nanomedicine-based treatments often resulted in the lack of, or the limited gain in, overall patient survival [8]. For instance, the first approved PEGylated liposomal doxorubicin formulations (Doxil^®^, Baxter Healthcare CorporationDeerfield, IL, USA and Caelyx^®^, Janssen Pharmaceutica NV, Turnhoutseweg, Beerse, Belgium) showed improvements in safety but not in efficacy compared to the standard therapies [9]. Moreover, although all the attempts to develop advanced nanosized drug delivery systems (DDSs) alternative to the conventional approved liposomal formulations, their clinical translation has been frequently hampered by several technical and cost challenges. Therefore, a serious skepticism towards the use of pharmacological nanocarriers (NCs) is growing in the scientific community [10,11,12].

However, such uncertainty seems to be somewhat overestimated. Indeed, the mentioned limitations highlight the poor understanding of tumor biology as a consequence of the incomplete predictability of the available preclinical models and the large heterogenicity in the patient population. Particularly, the relevance of the EPR effect, which was acknowledged as the “royal gate” in the DDS field, should be now reconsidered in the light of the inter- and intra-patient variability [13]. Additionally, deeper comprehension of the nano–bio interactions may point out new perspectives as well as indicate the most promising approaches to be pursued. Indeed, besides ameliorating the delivery of small chemotherapeutic agents to the tumor cells, new strategies are currently under investigation, including the possibility of exploiting nanoparticles for biologics administration and targeting or activating cellular populations different from the cancer cells (e.g., improving the immunotherapy efficacy) [13,14].

This review aims to disclose the current hurdles encountered in the clinical translation of nanotherapeutics that have been validated at the laboratory level, focusing on the products’ development as well as their biological fate after in vivo administration. We also discuss the nanomedicine impact in the oncology field and propose innovative strategies for maximizing their performance.

## 2. State of the Art in Nanomedicine Research

**Figure 1:**
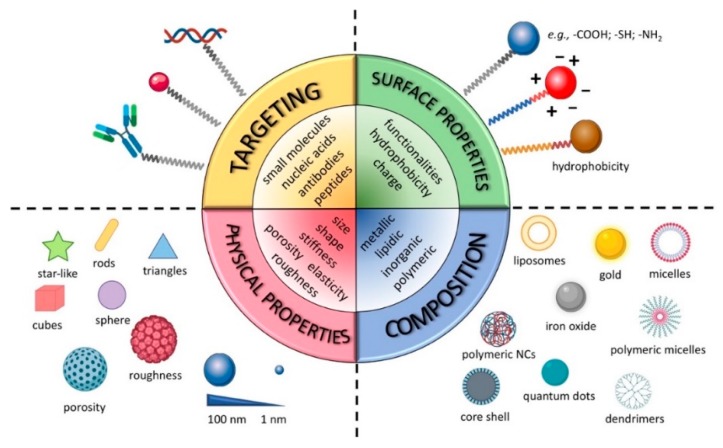
Tunable physical and chemical properties of nanocarriers (NCs).

The main purpose of this section is to give a general picture of the biological processes in which the NCs are involved, once administered in vivo, as well as their clinical implications. However, it is worth mentioning that the NCs’ fate and therapeutic outcome is strongly affected by their particular chemical composition and other specific structural features, including surface properties (e.g., charge and hydrophilic to hydrophobic ratio), general physical characteristics (e.g., size, shape, and stiffness) and functionalization (Figure 1).

### 2.1. Protein Corona

One of the main issues relating to the clinical translation of NCs is represented by the lack of comprehensive knowledge about the interaction between NCs and biological fluids. In particular, the high protein concentration of the physiological environment greatly affects the NCs’ biological behavior. Indeed, in response to the characteristics of the administered nanoparticles, endogenous proteins promptly adsorb on the surface, creating the so-called protein corona (PC). As established by several groups, this layer is highly dynamic, and its composition is strongly influenced by the biological milieu [15,16]. The protein adsorption impacts particle size, stability, surface properties, and defines the NCs’ biological identity and, therefore, their fate [13]. For instance, binding with opsonins (e.g., IgGs and complement proteins), as well as some conformational changes in the attached proteins, trigger NCs uptake by the mononuclear phagocyte system (MPS) [17]. Although in early studies, the PC relevance was confined to some undesirable effects, including NCs clearance and activation of the immune system, it is now believed that in several circumstances, PC also dictates the cellular uptake and intracellular localization of NCs [17,18,19]. While most studies involving nanoparticles as DDSs were originally intended to discourage the protein adsorption by surface coating with hydrophilic polymers (e.g., polyethylene glycol, PEG; zwitterions; carbohydrates; etc.), more recently, some innovative strategies have attempted to benefit from these spontaneous interactions. Such strategies may be accomplished by promoting the adsorption in vivo or by decorating in vitro the NCs surface with specific proteins, which decrease the MPS uptake and/or preferentially lead to a targeted delivery [20]. A recent example of the latter approach was the regulation of the PC formation by precoating the NCs with a HER2 affibody–glutathione–S–transferase fusion protein. This study demonstrated that the formation of a protein shield reduces the adsorption of serum proteins maintaining the selective targeting ability of the targeting molecule [21].

Considering the multitude of processes in which the PC is directly involved, it is of paramount importance to better understand the driving forces that lead to the PC formation and how it can be manipulated to predict the NCs’ fate after their administration in vivo. Nowadays, despite many efforts, a validated model to mimic the in vivo PC generation, as well as an affordable characterization method, are still missing.

### 2.2. Pharmacokinetics and Biodistribution

Several parameters affect the pharmacokinetics and biodistribution of NCs, including the administration route and NCs’ features, such as size, shape, surface, and mechanical properties. After systemic administration, the major clearance organs are lungs, liver, spleen, kidneys; the relationships between the biodistribution in such organs and the NCs properties are reported in Table 1. In general, for particles above the renal threshold (size > 5.5 nm) [22], the elimination is performed by tissue-resident macrophages, monocytes, and dendritic cells belonging to the MPS, although the role of endothelial cells has been recently reconsidered [23,24].

As already mentioned, several strategies have been developed to escape the MPS recognition, and the most established exploits antifouling polymer grafting (e.g., PEG) onto NCs surface. Thus, some clinical products (i.e., Doxil^®^ and Onivyde^®^, Les Laboratoires Servier Industrie, Route de Saran, Gidy, France) take advantage of PEGylation to facilitate the immunoevasion. However, the steric barrier created by this polymer does not specifically prevent the interaction with the MPS. Additionally, in some patients, phenomena such as complement activation, infusion reactions, and the production of PEG antibodies have been observed [31,32,33]. Alternative and more effective strategies are currently under investigation, including the functionalization with CD47 self-peptide and the use of engineered extracellular vesicles or cell membrane-coated particles [34,35]. All these approaches are intended to prolong the circulation time of NCs, although the extended half-life is not always associated with an improvement in the tumor accumulation. In this context, the drug release kinetics (Section 2.5), along with target accessibility (Section 2.3), have been demonstrated to significantly contribute to the treatment response.

### 2.3. Tumor-Specific Accumulation

**Figure 2:**
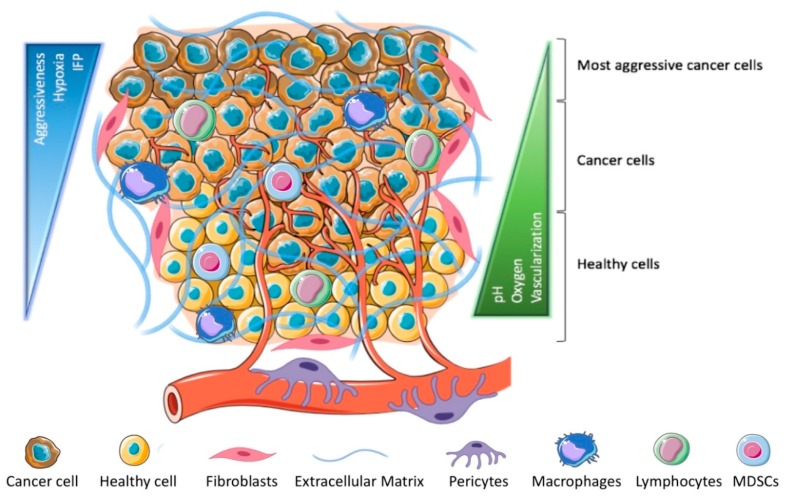
Tumor microenvironment. Tumor tissue is characterized by a high cellular density that hampers oxygen and nutrient perfusion. Accordingly, cancer cells are genetically and epigenetically heterogeneous, and those located far from the blood vessels: (1) favor an anaerobic metabolism that leads to the environment acidification; (2) are more resistant to pharmacological treatments because of their low division rate and genetic instability [42]. Fibroblasts and pericytes are responsible for tissue remodeling, while the immunosuppressive milieu hinders immune cell activity. NCs extravasation and penetration are mainly limited by solid stress and high interstitial fluid pressure, which in certain areas may reach values close to the aortic pressure [43].

A tumor mass consists of proliferating cancer cells and stromal cells (i.e., fibroblasts, immune, and perivascular cells), supplied with a dense extracellular matrix (ECM) and a tortuous and chaotic blood vessels’ network. The architecture and properties of this organ-like entity are illustrated in Figure 2. In addition to cancer onset and progression, the so-called tumor microenvironment (TME) is closely involved in tumor resistance to treatments [36,37]. The understanding of tumor biology is of utmost importance in nanomedicine also because TME was clearly demonstrated to minimize the NCs’ efficacy by opposing several barriers. First of all, the nanocomplex extravasation is required and generally associated with the EPR effect that characterizes the tumor region [38]. Actually, the discontinuous and fenestrated blood vessels, together with the poor lymphatic drainage, led to optimistic over-expectations in nanomedicine. However, differently from the experimental confirmations achieved from preclinical models, increasing evidence suggests that the great variability in the extent of the EPR effect can be found both among patients and tumor types [39]. Moreover, recent studies revealed that, besides the leaky blood vessels, endothelial transcytosis, as well as vascular bursts, enhance tumor permeability [40,41].

Once extravasated, NCs are expected to homogeneously distribute within the tumor area, but tissue penetration is strongly hampered by several obstructing factors, including the elevated interstitial fluid pressure (IFP) caused by inefficient lymphatic drainage and blood vessel compression, and solid stress associated with high cellular density and excessive production of ECM [6]. These factors restrict the NC action to the cells located in the perivascular space, hiding the more resistant cells placed in the hypoxia regions.

Therefore, the tumor accumulation can be considered as the major hurdle to the clinical translation and application of nanosized DDSs. For this reason, the TME modulation and the patient stratification have been recently proposed as strategies to improve the nanodrugs’ performance, as discussed below. In addition, an exhaustive understanding of the factors that guide the tissue penetration is extremely urgent. Size, shape, and surface chemistry have been identified as the major characteristics responsible for NCs diffusion inside the tumor mass [44,45]. Beside some universally accepted correlation, such as the inverse proportion between NC size and penetration ability, there are still controversial opinions about the impact of surface charge [46,47]. The functionalization of the NC surface with tissue and cell-penetrating peptides, such as the iRGD peptide, is a promising strategy to increase vascular and tissue permeability. Specifically, iRGD interacts with α_ν_ integrins on the endothelium, stimulating a proteolytic cleavage, and the subsequent binding of the released C-end-R peptide with neuropilin-1, ensuring both the tumor homing and tissue penetration [48]. Recent concerns about possible non-specific interactions of iRGD that might reduce the target accumulation of NCs could be overcome by triggering the penetrating peptide exposure in the TME following specific stimuli [49]. Another common approach to minimize the interaction between NCs and the ECM is surface PEGylation, as has been demonstrated in different tumor models, such as orthotopic brain and lung cancers [50,51]. However, a dense PEG layer may discourage interaction with target cells. Hence, the NC properties should be carefully optimized to balance their diffusion and target recognition capabilities.

### 2.4. Cellular Internalization

An effective cellular uptake has an impact on the therapeutic response, as many drugs are directed towards intracellular targets. Notably, an enhanced internalization is crucial for improving the activity of both biologics and small molecules, as it allows poor cellular permeability to be overcome, and the multidrug efflux transporters to be bypassed, respectively [52,53]. The most common approach to increase the NCs uptake is “active” (i.e., molecular) targeting. This strategy aims at improving the selectivity of NCs toward the target cells by decorating their surface with affinity ligands that recognize receptors specifically overexpressed on tumor vasculature or tumor cells [54]. However, the molecular forces that drive ligand–receptor binding only extend over 0.3–0.5 nm [55]. Thus, to promote tumor retention and increase cellular uptake, an efficient NC extravasation is needed [12]. It should be noted that the NC functionalization is expected to alter their physical–chemical properties, affecting the MPS uptake as well as the intratumoral penetration [31]. Moreover, the targeting receptor should be carefully selected, taking into account its preferential tumor expression compared to normal tissues and immune cells, as well as its capability to internalize the NCs upon interaction. A big concern for active targeting success is posed by cancer cell heterogeneity: indeed, it is well known that the cancer cells’ epigenetic diversity leads to different expression levels of the targeting receptor [10]. On the other hand, cellular internalization is performed by receptor-mediated endocytosis, which usually leads to NC degradation. In this context, for all the therapeutics that are susceptible to lysosomal digestion (e.g., biologics), an efficient endosomal escape is essential to preserve drug efficacy [56]. Different strategies have been proposed to promote this event, such as membrane fusion, osmotic rupture, particle swelling, and membrane destabilization [57]. Despite these efforts, the proportion of NCs that actually perform the endosomal escape remains extremely low, and thus, more efficient or alternative approaches are demanded [58]. For instance, Rotello and coworkers proposed an endosomal-free cytosolic delivery based on the direct fusion between the nanoassemblies and the plasma membrane [59], whereas Gong and colleagues have recently developed a polymeric coating, termed nanocapsule, with an improved endosomal escape compared with commercial agents [60]. All these concerns underline that, although targeted nanomedicine was proposed as a magic bullet for cancer treatment, its clinical relevance still needs to be validated. Indeed, despite the superiority of the active over the merely passive targeting demonstrated in preclinical models, at present, none of these nanodrugs have been approved [8]. Therefore, increased awareness of the molecular mechanisms governing active targeting is imperative, considering that common belief on nanoparticle-biosystem interactions do not always allow for reliable predictions. For instance, as demonstrated by Colombo et al., maximizing the number of targeting moieties on the NC is indeed expected to improve the molecular targeting in vitro but does not necessarily result in superior therapeutic performance in vivo [61].

### 2.5. Drug Delivery and Release

In a drug delivery framework, the nanoformulation is intended to enhance the drug protection and permeability, to extend the therapeutic agent half-life, to improve the drug solubility and/or increase its therapeutic index [62]. As already stressed above, different types of drugs, including both small molecules and biologics, can benefit from nanoscale DDSs in enhancing their therapeutic efficacy. Indeed, NCs may broaden the spectra of the administered drugs when they are small molecules, whereas they may overcome the drawbacks associated with large, hydrophilic, and delicate biological molecules improving their availability or helping them to cross the biological barriers [63,64]. However, it is worth mentioning that the release performance of these DDSs should be carefully investigated, and the NCs’ design possibly optimized. Especially for long-circulating NCs, minimizing the premature drug release is fundamental to improve its therapeutic outcome [65]. Additionally, new nanoparticle-based classes of DDSs have been recently developed to precisely control the drug release in response to specific conditions, such as the stimuli-responsive NCs that will be discussed further below (Section 5.1.2) [13,43].

## 3. Controversies Around Clinical Translation of Cancer Nanomedicines

The first nanomedicine that received clinical approval was the PEGylated liposomal formulation of doxorubicin in 1995 (Doxil^®^/Caelyx^®^). Since then, 15 nanodrugs have been developed and tested for cancer treatment and have entered the market (Table 2). Doxil^®^/Caelyx^®^, together with the albumin-based formulation of paclitaxel (commercialized by Celgene corporation under the Abraxane^®^, Summit, NJ, USA trademark), currently represents the top-selling nanomedicines in 2018, accounting for $252 M and $950 M, respectively [8]. The liposomal doxorubicin formulations demonstrated a different drug distribution compared to standard treatments, limiting the cardiotoxicity induced by anthracyclines [66]. The new therapeutic index achieved broadened the spectrum of treatable candidates and improved patient compliance. In turn, Abraxane^®^ strongly enhanced paclitaxel tolerance, allowing drug administration without the use of toxic solubilizing surfactants (e.g., castor oil—cremophor EL^®^). Notably, clinical studies have demonstrated a significant increase in the maximum tolerated dose as well as shorter infusion time of Abraxane^®^ compared to a cremophor EL^®^-based formulation [67].

However, despite the important advantage of these nanomedicines in terms of safety, the treatment efficacy did not increase as expected. So far, most approved nanodrugs exhibited only a moderate impact on overall survival as compared to relevant standard therapies [8]. Among the nano-based products under clinical investigation, some of them aim to ameliorate the cancer treatment performance by means of active targeting (e.g., BIND-014) and stimuli-responsive drug release (e.g., ThermoDox) [68,69]. Nevertheless, the low efficacy still represents the main hurdle to the nanodrugs’ clinical translation. In particular, among the 94% successful phase I trials, only 14% concluded phase III with positive outcomes [8]. This disappointing efficacy is likely due to multiple factors, such as an incomplete knowledge about the nano–bio interactions (Section 2) and poor reliability of the existing preclinical models. Lack of reliable disease models is particularly disappointing, as mouse tumor models fail to recapitulate the complexity of human tumors mainly because of their large size, the limited cancer cell heterogenicity, the exaggerated EPR effect, and general immunodeficiency [70].

Additional drivers behind the modest clinical translation of nanomedicines are technical and cost challenges in product manufacturing and scale-up. Several clinical trials were terminated or delayed due to unaffordable financial burdens. Indeed, the development of next-generation products other than the conventional liposomal formulations requires huge investments and poses serious issues about the process reproducibility [8,9]. Overall, the large pharma companies, the only entities that can afford such a prohibitive expense, are discouraged from supporting the clinical investigation because of the low perceived success chances. Therefore, it is of utmost relevance to consider that product clinical outcomes and funding availability are closely related since pharmaceutical companies and the healthcare system are more prone to invest if the improvement in treatment efficacy is significant [8].

## 4. Is It Still Reasonable to Invest in Cancer Nanomedicine?

**Figure 3:**
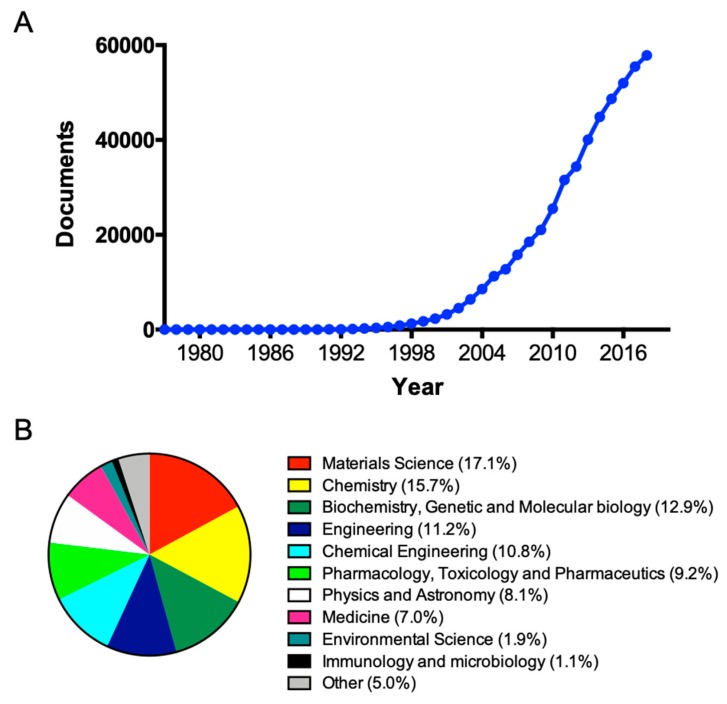
(**A**) Publication peak of “nanoparticles” related papers; (**B**) Subject areas of “nanoparticles + cancer” papers. Elaborated data are derived from the Scopus research tool.

As already mentioned, nanotechnology has attracted great interest in cancer treatment due to the unique physiochemical properties of nanostructures that can be exploited for diagnostic and therapeutic purposes. Searching “Nanoparticles” on Scopus.com, a publication peak is notable in 2018 with 57,434 documents (Figure 3A) and 22.7% (16,395 documents) of them related to cancer treatment and diagnosis. However, focusing on the subject area (Figure 3B), most of these works were reported in materials and chemistry-related journals, whereas only 17.3% were published in journals referring to the medical research area (including pharmacology, toxicology, and pharmaceutics; medicine; immunology and microbiology).

Such publication distribution corroborates the assumption that cancer nanomedicine is more focused on a “formulation-driven” rather than a “disease-driven” approach. Many researchers have pointed out that this one, together with overgeneralizing drug-targeting/delivery concepts and overselling preclinical studies, are the main causes of the suboptimal clinical translation of cancer nanomedicine [71]. In fact, even if the cancer nanomedicine research comprises different types of materials used for synthesizing NCs, only lipidic, one protein-based, a few polymeric and inorganic nanovehicles have been approved for marketing (Table 2) [72]. These considerations highlight the current limitation of nanomedicine in cancer treatment, leading the scientific community to ask if it is still reasonable to continue to invest in this field. As it can be inferred by Section 2 of this review, ready after administration NCs have to face many hurdles that can reduce therapeutic/targeting/accumulation efficiency. However, it is worth mentioning that both successes and failures have contributed to change the focus of studies to better understand the interaction between NCs and numerous cancer biological mechanisms, triggering new discoveries and future ambitions [9,13,73]. To make some examples, the emerging concerns regarding the real efficacy of the EPR effect in humans forced scientists to go in-depth in defining human TME characteristics to develop NCs with different size, shape, and surface properties to increase their penetration into the tumor mass [13]. Otherwise, the modulation of TME components (e.g., vasculature, ECM) is another interesting point of view to improve the delivery of nanomedicine to solid tumors [12,74].

Another crucial step in cancer nanomedicine is the relationship between the treatment efficacy and the immune system response. This vast branch of nanomedicine offered the opportunity to understand that (1) the available animal models are largely unsuitable, because usually scientists make use of immunodeficient mice, and (2) NCs can be sequestered or opsonized by immune cells [75,76]. In particular, tumor-associated macrophages (TAMs) are one of the primary biological barriers in cancer tissue invariably encountered by NCs, so that scientists tried to exploit this “disadvantage” using TAMs as a reservoir of nanovehicles, to increase the site-specific drug release [13,77]. Another interesting progress is the design of NCs that can avoid phagocytosis through the modification of their surfaces (e.g., with CD47) [78] or that can modulate the polarization and activity of macrophages [79,80]. In addition, studies about the numerous molecular targets found to develop specific active targeted NCs brought huge knowledge. Even if the real efficacy of active targeting in cancer affected patients is still in debate, this kind of approach allowed improvement in the awareness about the molecular characteristics of different cancer types [10,72,81]. Moreover, the evidence that NCs could be entrapped inside endosomes and/or lysosomes led the scientists to study this mechanism developing NCs able to reach the cell cytosol by clever strategies, including direct fusion with the plasma membrane [59] or by performing enhanced endosomal escape [82,83,84]. The use of engineered NCs can bring other opportunities, such as the encapsulation of poorly soluble drugs [85,86,87], as well as the delivery of biologics improving their bioavailability, permeability, and stability in the biological environment [53,72]. Thanks to NCs, it is possible to use drugs already accepted by clinical trials, opening the chance to administer them by different routes (i.e., topic [88], oral [89], and inhalation [90] rather than intravenous). This is a relevant point in locally administered therapies because this approach could re-establish the importance of active targeting by decreasing the number of physical and biological barriers that NCs need to overcome.

All these observations justify the ongoing enthusiasm which believes in nanomedicine that will lead to further investment in this field regardless of the unsatisfactory success rate hitherto achieved. However, it is urgent to reduce the gap between the huge number of published papers and the poor clinical outcome of these technologies. First, a sincere effort in the establishment of more clinically relevant models is required. In this context, 3D cultures (e.g., organoids and spheroids) have been proposed as an alternative to 2D cultures for in vitro purposes [91,92], whereas innovative in vivo models, such as patient-derived xenografts and genetically engineered mice, aim to recapitulate the complexity of human tumors [93]. Interestingly, the chicken chorioallantoic membrane (CAM) is emerging as a less time-consuming and a cost-effective alternative to the conventional mouse models [94]. Moreover, it is equally necessary to improve the research data collection to make them as informative as possible. In this regard, Caruso et al. suggested standardization of bio–nano experimental investigations [95], although, among the scientific community, the debate about aspects that need to be improved is still open [96]. Finally, other key points have been suggested by the European Technology Platform for Nanomedicine (ETPN) Agenda to ameliorate the clinical translation: (1) to change from a “formulation-driven” to a “disease-driven” approach considering the influence of tumor pathophysiology in the clinical outcome and/or focusing on unmet medical needs; (2) to facilitate and increase the dialogue between all the scientific disciplines that play a role in cancer nanomedicine; (3) to consider the balance between benefit for patients and cost constraints for the healthcare system; and (4) to sustain competitiveness of the healthcare economy at the global market [73].

## 5. Outlook on Future Strategies

Many factors still limit nanomedicine clinical translation and application. However, the presence of several currently active research areas demonstrates that there is still a lot of interest in filling this gap. Accordingly, this section aims to discuss the most promising strategies.

### 5.1. Strategies to Enhance Tumor Accumulation

Among the above-mentioned issues that hampered the clinical translations of nanomedicines, including overcoming biological barriers, increasing bioavailability, and circulation time of nanodrugs, improving active targeting, etc., enhancing tumor accumulation remains a primary objective. The following approaches are envisioned to allow researchers to step forward.

#### 5.1.1. Priming of the TME

Since several barriers in the TME prevent the nanomedicines delivery, many attempts, extensively reviewed elsewhere [97,98,99], have been made to improve the EPR effect by lowering the solid stress as well as the IFP. Among them, the normalization of the abnormal tumor vasculature aims to restore a more physiological condition, reducing the vessel leakiness, strengthening the structure of the basement membrane, and improving the coverage of pericytes. Although anti-angiogenic therapy may appear to prevent the tumor accumulation, it has been demonstrated that the vasculature is still permeable to relatively small NCs (20–40 nm), and the significant reduction in the IFP causes increased tumor retention [100,101]. The normalization process is usually achieved by inhibiting pro-angiogenic effectors, such as the VEGF (e.g., through bevacizumab) or PDGF (e.g., using imatinib) [100,101]. Another reported approach is the reduction of solid stress by inducing the tumor cells’ apoptosis. Indeed, the rapid cancer cell proliferation causes a compression of lymphatic and blood vessels promoting hypoxia, inflammation, immunosuppression, and metastasis, also representing an obstacle for drug penetration [102]. In this context, it has been observed that paclitaxel tumor priming reduces cell density and IFP, improving the penetration of several NCs, as observed for doxorubicin-loaded liposomes and lipid siRNA complexes [103,104,105]. Furthermore, ECM degradation has been proposed to alleviate solid stress and enhance NCs intratumoral accumulation. Notably, the use of collagenases, relaxin, and hyaluronidases have been explored for this purpose, and their association with chemotherapeutics is already under clinical trials [106,107,108]. Although efficient in promoting the tumor accumulation, the clinical application of such strategies is hampered by safety concerns, high costs, and the intrinsic instability of biologic drugs. To tackle these limitations, delivery improvement of these agents and/or the use of alternative cost-effective and well-tolerated small molecules (e.g., the already available Celecoxib) are under investigation [109]. Additionally, another relevant drawback of TME alteration is the possible promotion of tumor progression and invasiveness [31].

In addition to all the above, NCs biodistribution can be ameliorated by interfering with the MPS activity. Notably, tumor homing can be improved by saturating the main clearance organs with decoy NCs or by inhibiting the MPS uptake. Indeed, considering the negligible fraction of NCs that reach the tumor after administration, even small changes in clearance organs accumulation could significantly affect the therapeutic outcome [31,110,111,112].

#### 5.1.2. Nanocarriers Engineering

In addition to tumor priming, NC engineering may represent a valid strategy for improving nanomedicine performance. Among the reported approaches, stimuli-responsive NCs play a prominent role. These nanoformulations exploit specific endogenous or exogenous stimuli that trigger drug release, specifically within the tumor tissue. In the first case, NCs responsive to acidic pH, hypoxic environment [113,114,115], overexpression of tissue remodeling enzymes (e.g., MMP2-9) [116] or the high intracellular concentration of glutathione demonstrated an increase in the cargo therapeutic efficacy and safety [117]. On the other hand, magnetic, thermo-, electric-, light- and ultrasound-sensitive materials may be employed for nanodrugs development to promote the intratumoral drug delivery [118,119,120]. In this context, several products, such as thermosensitive liposomes (Thermodox), enzyme activated polymeric NCs (Opaxio), as well as magnetic nanoparticles (MTC-Dox), are currently under clinical investigation or approved [43]. Moreover, the combination of different stimuli has recently been proposed to further improve the efficacy of nanoscale DDSs for cancer treatment [43]. In general, the concept of multifunctional vectors is slowly establishing together with a wider comprehension of nano–bio interactions. In particular, novel systems capable of modifying their proprieties in a spatiotemporal way (multistage DDSs) have been developed. For instance, relatively big NCs (<200 nm) can respond to specific stimuli releasing small particles (5–15 nm) able to deeply penetrate into the tumor tissue [121,122]. Alternatively, in multi-layered NCs, the external shell (e.g., PEGylated responsive materials) is expected to change in proximity to a tumor, exposing the hidden penetrating peptides or targeting agents [123,124,125]. Despite the interesting results, it remains questionable whether increasing the complexity of NCs could excessively hinder their clinical translation. In light of the above consideration, another option may be using nature-inspired NCs composed of biological materials, such as proteins (e.g., albumin, lipoproteins, ferritin) or cellular-derived membranes (e.g., cancer cells, platelets, erythrocytes, and leukocytes) [126]. These materials are generally well-tolerated, less recognized by MPS, and are eventually able to increase tumor targeting. For instance, ferritin-based NCs showed an intrinsic tumor homing as well as an improved performance compared to the liposomal formulation, when loaded with doxorubicin [127]. Another pioneering approach exploited the use of engineered leukocytes membrane to enhance the NCs’ accumulation in the proximity of inflamed tumor tissues [128].

#### 5.1.3. Optimizing the Administration Route

Currently, most of the nanomedicines are intravenously injected, but to increase the NCs potential and adopt a more “disease-driven” approach, alternative administration routes might be considered. For instance, the local administration of drug-loaded NCs could perform better than the systemic one because it could reduce the off-target toxicity as well as increase the tumor accumulation bypassing the physiological barriers [54]. This strategy is particularly recommended for non-metastatic tumors or when surgery is contraindicated [54]. As recently reviewed, for lung cancer therapy, the pulmonary route has been explored to improve drug delivery [129]. The local administration proved to be effective even in glioma models where drug-loaded NCs could show a safer toxicity profile compared to the free molecule [50]. Furthermore, some clinically approved products take advantage of local administration: Hensify^®^ (Nanobiotix, Rue de Wattignies, Paris, France) enhances the performance of radiotherapy in advanced soft tissue sarcoma, whereas intracranially injected iron oxide nanoparticles (Nanotherm^®^) efficiently induced hyperthermia in glioblastoma treatment [130,131]. In addition, non-conventional systemic administration routes may be investigated to specifically accumulate drugs to cellular or tissue targets. Particularly, non-invasive intranasal administration may be exploited for the nose-to-brain delivery, circumventing the first passage in the liver and the blood–brain barrier, thus increasing the fraction of drug at the target site [132,133]. On the other hand, intraperitoneally injection proved to be effective in targeting circulating macrophages, which, once repolarized, exhibited inherent tumor tropism [134]. Finally, as demonstrated by the recent approval of DHP107 (Liporaxel^®^, DAE HWA Pharm, Seoul, Korea) the oral route has been investigated for increasing patient compliance and reduce the therapy costs [135].

### 5.2. Nanoimmunology and New Targets

Although classical nanotherapies are directed towards cancer cells, innovative approaches rely on targeting alternative cellular components. Considering the increasing role of cancer immunotherapy, not surprisingly, most of these new targets belong to the immune system. As extensively reviewed, many approaches have been investigated to ameliorate the impact of cancer immunotherapy through the use of nanomedicine [14,136,137]. Here we focus on those that directly modulate the activity of particular cellular mediators, such as tumor-associated macrophages (TAMs), myeloid-derived suppressor cells (MDSCs) and regulatory T cells (Treg) [138]. TAMs are usually characterized by a high M2/M1 ratio, leading to an immunosuppressive environment that promotes tumor progression [139]. The three main strategies, which target TAMs are (1) repolarization of M2 in M1; (2) abolishment of macrophage recruitment via cytokines inhibition; (3) eradication of M2 cells [138,140]. Notably, the selectivity towards M2 can be achieved by targeting the overexpressed mannose receptor [141]. Moreover, the targeting of CD44, along with the intraperitoneal injection, leads to the macrophage-specific delivery exploitable for the repolarization strategy [134].

Other currently investigated targets are the MDSCs, immature cells that contribute to tumor progression by releasing immunosuppressive cytokines [138]. Nanomedicine aims to promote their differentiation into mature cells [142,143], as well as interfere with MDSCs accumulation/activity, by improving the drug delivery [144].

Similarly, Tregs mediate the immunosuppression by inhibiting the activation and expansion of effector T cells, and their downregulation could be ameliorated by the use of NCs. In particular, Tregs can be actively targeted by using their specific markers, such as glucocorticoid-induced Tumor Necrosis Factor Receptor-related protein (GITR), or neuropilin-1 receptor by binding of tLyp1 peptide [145,146]. However, this therapeutic strategy needs to be further validated because Tregs instability could be associated with the onset of autoimmune disorders [147].

In addition to immune cells, cancer-associated fibroblasts (CAFs) have been recently identified as candidates for antitumoral therapies because they are responsible for both immunosuppression and TME reorganization [148,149]. Alternatively, the TME can be directly modulated by NCs. Indeed, the reduction of tumor hypoxia, the restoration of physiological pH, and the inhibition of immunosuppressive soluble mediators impair the tumor progression improving the outcome of current therapies [150].

### 5.3. Companion Diagnostic

A promising strategy to improve nanomedicine efficacy is the companion diagnostic, which refers to a stratification of patients based on tumor characteristics. Different strategies are currently under investigation based on the use of biomarker profiles and imaging data. The first aims to identify circulating proteins associated with the TME and positively correlated to the EPR effect. For instance, the ratio of MMP9 to the tissue inhibitor of metalloproteinase 1, the collagen content in the capillary walls, and some angiogenesis markers have been shown to predict the EPR entity [13]. On the other hand, radio-labeled and ferumoxytol-loaded NCs have been adopted to monitor their biodistribution by non-invasive techniques (e.g., Single Photon Emission Computed Tomography or Photon Emission Tomography and magnetic resonance imaging, respectively) [13]. The final goal is selecting patients that present the highest probability to positively respond to a specific therapeutic treatment [31]. However, to reach a real utility in clinics, these approaches need to be further validated by accurate correlative studies, defining a clear set of parameters and criteria able to predict the therapeutic outcome [9].

## 6. Conclusions

The unique attributes of nanoparticles allow clinicians of the 21st century to design innovative therapeutic strategies for use as monotherapies or to be combined with existing chemotherapeutic treatments or conventional radiotherapy. The recent advances achieved by researchers in the development of tumor-targeting NCs together with a faster data collection deriving from the study of their communication with the biological milieu has generated optimistic expectations for the rapid translation of this basic research into the clinical practice with immediate benefits for oncology patients. However, only a few nanodrugs have actually reached the marketplace and are now approved by the FDA or EMA for specific cancer treatments. This transitory failure has raised some criticisms on the real effectiveness of nanomedicine so that the huge amount of resources dedicated to the research in this field in the last decade has been questioned. This review highlights the main challenges that the scientific community, assisted by the health system and industry, should face in a virtuous joint effort aimed to bring the new discoveries to an established practice that would allow the regulatory bodies to accelerate the process toward the bedside (Section 4). A well-standardized toolkit for the physicochemical, pharmacological, and immunological characterization of all newly developed nanodrugs should be defined before they can be approved for use in humans. The distribution of nanoparticle size, uniformity, surface coating, colloidal stability, and reproducibility from batch to batch also needs to be accurately regulated. Recently, attempts to overcome such barriers to the progression of nanooncology have suggested the definition of a “minimum information standard” for experimental protocols associated with the investigation of the nano–bio interface, leading to the so-called MIRIBEL (minimum information reporting in bio–nano-interaction) paradigm [95]. The collection of three standard categories should be satisfied to fulfill the minimal requirement for good practice in nanomedicine, including appropriate material characterization, biological characterization, and details of experimental protocols. Furthermore, standardized assays for the assessment of short-term and long-term toxicity of nanoparticles will also need to be defined in 2D/3D cell cultures and animal models before approval for clinical trials. Eventually, the cross-fertilization of nanotechnology with recent progress in advanced immunotherapies, together with a renowned knowledge of the impact of environmental factors (e.g., microbiota) on cancer, is expected to trigger a new spur in nanomedicine discovery [151,152]. This entails that nanomedicine researches in the future will be invited to move from a limited “formulation-driven” approach to a preferential “disease-driven” setting, leading to a new era of nanooncology.

## Figures and Tables

**Table 1:** Properties-dependent clearance of nanocarriers (NCs).

	Biodistribution Profiles in Clearance Organs
SIZE	Renal excretion is particularly relevant for NCs below the threshold value (ca. 5.5 nm) [25]. MPS clearance is operated by liver>spleen>lung phagocytes. However, the spleen and lung fraction increase with the particle size: NCs > 150 nm are more prone to splenic filtration, while lung accumulation is particularly promoted when the NCs size is close to the micromillimeter range, or they aggregate [26,27].
SURFACE CHARGE	It is generally accepted that positively charged NCs are more rapidly sequestered by MPS than negative and neutral NCs due to the highly-dense coating of serum proteins formed on the administration [27]. However, the surface hydrophobicity, as well as the charge density, strongly influences the elimination rate [28].
SHAPE	NCs’ shape determines the movement in blood circulation and the organ-specific biodistribution [25]. Spherical NCs presented the longest circulation time, while rod-, disc-, cage- particles exhibited an increased splenic and hepatic accumulation compared with spherical counterparts [29].
STIFFNESS	Due to the intrinsic deformability, soft NCs have prolonged circulation lifetimes and reduced splenic accumulation when compared with rigid NCs [30].

**Table 2:** Clinically approved cancer nanomedicines [8,31].

Product Name	Composition	Indications	First Approval
Doxil/Caelyx	PEGylated liposomal doxorubicin	Myeloma, Kaposi’s sarcoma, breast, and ovarian cancer	Approved in the US (1995)
DaunoXome	liposomal daunorubicin	Kaposi’s sarcoma	Approved in the US (1996)
Myocet	liposomal doxorubicin	Breast cancer	Approved in Europe/Canada (2000)
Abraxane	albumin-bound paclitaxel	Breast, non-small-cell lung, and pancreatic cancer	Approved in the US (2005)
Lipusu	liposomal paclitaxel	Breast and non-small-cell lung cancer	Approved in China (2006)
Oncaspar	L-asparaginase conjugate	Acute lymphoblastic leukemia	Approved in the US (2006)
DepoCyt	liposomal cytarabine	Lymphoma, Leukemia	Approved in the US (1999)
Genexol-PM	paclitaxel micellar	Breast, non-small-cell lung, ovarian, and gastric cancer	Approved in Korea (2007)
Mepact	liposomal mifamurtide	Osteogenic sarcoma	Approved in Europe (2009)
NanoTherm	Iron oxide nanoparticles	Brain tumors	Approved in Europe (2011)
Marqibo	Liposomal vincristine sulfate	Acute lymphoblastic leukemia	Approved in the US (2012)
ONIVYDE	liposomal irinotecan	Advanced pancreatic cancer	Approved in the US (2015)
DHP107	paclitaxel lipid nanoparticles (oral administration)	Gastric cancer	Approved in Korea (2016)
Vyxeos	liposomal daunorubicin and cytarabine	High-risk acute myeloid leukemia	Approved in the US (2017)
Apealea	paclitaxel micellar	Ovarian, peritoneal, and fallopian tube cancer	Approved in Europe (2018)
Hensify	hafnium oxide nanoparticles	Locally-advanced soft tissue sarcoma	Approved in Europe (2019)
